# Newborn Screening for Presymptomatic Diagnosis of Complement and Phagocyte Deficiencies

**DOI:** 10.3389/fimmu.2020.00455

**Published:** 2020-03-17

**Authors:** Mahya Dezfouli, Sofia Bergström, Lillemor Skattum, Hassan Abolhassani, Maja Neiman, Monireh Torabi-Rahvar, Clara Franco Jarava, Andrea Martin-Nalda, Juana M. Ferrer Balaguer, Charlotte A. Slade, Anja Roos, Luis M. Fernandez Pereira, Margarita López-Trascasa, Luis I. Gonzalez-Granado, Luis M. Allende-Martinez, Yumi Mizuno, Yusuke Yoshida, Vanda Friman, Åsa Lundgren, Asghar Aghamohammadi, Nima Rezaei, Manuel Hernández-Gonzalez, Ulrika von Döbeln, Lennart Truedsson, Toshiro Hara, Shigeaki Nonoyama, Jochen M. Schwenk, Peter Nilsson, Lennart Hammarström

**Affiliations:** ^1^Division of Clinical Immunology and Transfusion Medicine, Department of Laboratory Medicine, Karolinska University Hospital Huddinge, Stockholm, Sweden; ^2^Division of Affinity Proteomics, Department of Protein Science, KTH Royal Institute of Technology & SciLifeLab, Stockholm, Sweden; ^3^Department of Laboratory Medicine, Section of Microbiology, Immunology and Glycobiology, Lund University, Lund, Sweden; ^4^Clinical Immunology and Transfusion Medicine, Region Skåne, Lund, Sweden; ^5^Research Center for Immunodeficiencies, Pediatrics Center of Excellence, Children's Medical Center, Tehran University of Medical Sciences, Tehran, Iran; ^6^Department of Immunology, School of Medicine, Tehran University of Medical Sciences, Tehran, Iran; ^7^Immunology Department, Vall d'Hebron Research Institute, Hospital Universitari Vall d'Hebron, Universitat Autònoma de Barcelona, Barcelona, Spain; ^8^Pediatric Infectious Diseases and Immunodeficiencies Unit, Vall d'Hebron Research Institute, Hospital Universitari Vall d'Hebron, Universitat Autònoma de Barcelona, Barcelona, Spain; ^9^Immunology, Hospital Universitari Son Espases/Institut d'Investigació Sanitària Illes Balears, Palma, Spain; ^10^Royal Melbourne Hospital, Melbourne, VIC, Australia; ^11^The Walter and Eliza Hall Institute of Medical Research, Melbourne, VIC, Australia; ^12^Department of Microbiology and Immunology, Sint Antonius Hospital, Nieuwegein, Netherlands; ^13^Department of Immunology, Hospital San Pedro de Alcántara, Cáceres, Spain; ^14^Departamento de Medicina, Hospital La Paz Institute for Health Research (IdiPAZ), Universidad Autónoma de Madrid and Complement Research Group, Madrid, Spain; ^15^Primary Immunodeficiencies Unit, Department of Pediatrics, University Hospital 12 de Octubre, Research Institute Hospital 12 Octubre (I+12), Madrid, Spain; ^16^Immunology Department, University Hospital 12 de Octubre, Research Institute Hospital 12 Octubre (I+12), Madrid, Spain; ^17^Fukuoka Children's Hospital, Kyushu University, Fukuoka, Japan; ^18^Department of Pediatrics, National Defense Medical College, Saitama, Japan; ^19^Department of Infectious Diseases, Institute of Biomedicine, Sahlgrenska Academy, University of Gothenburg, Gothenburg, Sweden; ^20^Departments of Infectious Diseases, Central Hospital, Kristianstad, Sweden; ^21^Division of Metabolic Diseases, Department of Laboratory Medicine, Karolinska Institutet, Karolinska University Hospital Solna, Stockholm, Sweden

**Keywords:** primary immunodeficiency, complement deficiencies, phagocytic disorders, presymptomatic diagnosis, newborn screening, dried blood spot, protein profiling

## Abstract

The clinical outcomes of primary immunodeficiencies (PIDs) are greatly improved by accurate diagnosis early in life. However, it is not common to consider PIDs before the manifestation of severe clinical symptoms. Including PIDs in the nation-wide newborn screening programs will potentially improve survival and provide better disease management and preventive care in PID patients. This calls for the detection of disease biomarkers in blood and the use of dried blood spot samples, which is a part of routine newborn screening programs worldwide. Here, we developed a newborn screening method based on multiplex protein profiling for parallel diagnosis of 22 innate immunodeficiencies affecting the complement system and respiratory burst function in phagocytosis. The proposed method uses a small fraction of eluted blood from dried blood spots and is applicable for population-scale performance. The diagnosis method is validated through a retrospective screening of immunodeficient patient samples. This diagnostic approach can pave the way for an earlier, more comprehensive and accurate diagnosis of complement and phagocytic disorders, which ultimately lead to a healthy and active life for the PID patients.

## Introduction

Primary immunodeficiencies (PIDs) are a group of inherited disorders caused by defects in different components of the immune system. PIDs may be associated with severe clinical outcomes if left undiagnosed or untreated (1). However, it is uncommon to consider PIDs before the manifestation of severe clinical symptoms. Recently, novel newborn screening assays have been explored to enable early clinical intervention and disease management, beginning as early as the presymptomatic stage at birth. Newborn screening programs based on the use of dried blood spot (DBS) samples have revolutionized public healthcare by detecting disorders such as phenylketonuria (PKU) during the first few days of life (2). Further developments of this strategy introduced mass spectrometry (MS)-based screening with improved sensitivity, specificity and capacity (2). Most recently, DNA-based screening methodologies have been developed to detect severe PIDs with defects in adaptive immunity (2). PCR-based screening is being applied for diagnosing a subset of life-threatening PIDs, including severe combined immunodeficiency (SCID) and X-linked agammaglobulinemia (XLA), through the measurement of episomal excision products of lymphocyte receptors in DBS samples (3). Selected countries have successfully implemented SCID-screening and several additional countries worldwide are considering to include it in their national screening programs (2).

Here, we present a newborn screening based on protein profiling that broadens the diagnosis to cover other severe forms of PIDs with innate immunity defects. The screening enables parallel diagnosis of 22 disorders due to defects in the complement system or phagocytic function prior to the onset of clinical symptoms. Complement deficiencies represent 1–29% of all PID cases, (4) giving a cumulative prevalence of 1 in 20,000 live births (5), and phagocytic disorders encompass 5–29% of PIDs, occurring at a prevalence of 1 in 250,000 live births (1, 6). However, due to the lack of comprehensive and pre-symptomatic diagnosis, the prevalence rate of these PIDs is markedly underestimated (7). Accurate and early diagnosis is vital as complement deficiencies and phagocytic disorders are associated with numerous immunological complications. Complement deficiencies cause recurrent and persistent infections in the upper respiratory tract, recurrent pneumococcal and meningococcal infections, hereditary angioedema (HAE), autoimmune complications, and renal failure due to atypical hemolytic uremic syndrome (aHUS) and glomerulonephritis (GN) (8). Severe congenital neutropenia (SCN) and chronic granulomatous disease (CGD) are characterized by low granulocyte counts or defects in the nicotinamide adenine dinucleotide phosphate (NADPH) oxidase pathway, leading to conditions such as delayed wound healing, deep organ infections, and abscess formation. These infections may become severe or even lethal, and might lead to development of malignancies or bone marrow failure (6). Early diagnosis of such disorders allows immediate clinical intervention and prevention of severe complications. Moreover, in the case of phagocytic diseases, it might allow for early stem cell transplantation, with a future prospect of gene therapy.

**Figure 1:**
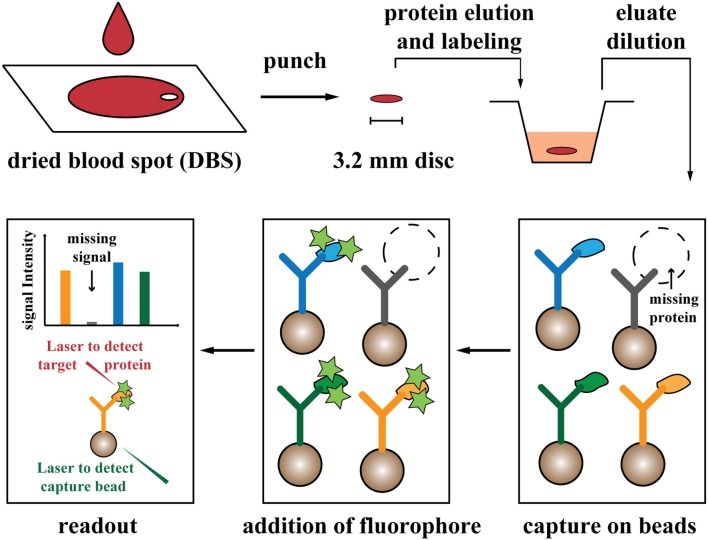
Schematic view of the screening procedure. Drops of blood (~70 μL) are dried on filter paper. Proteins are eluted and labeled from a punched disc, equal to a 4.5% fraction of one blood drop. After further dilution (1:500 dilution of blood), proteins are captured on the designed antibody-coupled bead array, and signals are analyzed with fluorescent-based readout. Stars illustrated fluorescent label. Two laser beams are applied for detection of beads (capture antibodies) and associated fluorescent signal (from labeled target proteins).

Currently, complement functional assays, nephelometry, immunoprecipitation (IP), enzyme-linked immunosorbent assay (ELISA), complete blood count and flow-cytometric cellular characterizations are applied for diagnosis of complement and phagocytic disorders (5). All these methods are low-throughput, hardly adaptable to population-scale performance, and can be costly and labor-intensive to test for several disorders in one sample. Moreover, the small quantity of blood material in DBS significantly limits the total number of possible tests on each sample (9). Accordingly, an alternative diagnostic method that enables multiple parallel tests and requires minimal sample volume would greatly improve the clinical procedures, particularly in newborn screening. Considering the disease mechanisms of complement and phagocytic defects that involve the absence, or expression/structural alteration of a functional protein, protein profiling of blood samples could provide a fundamental diagnostic tool (10). Here, we developed a newborn screening assay based on suspension bead array technology for parallel profiling of the main reported disease-associated proteins in the complement cascade, phagocytosis and respiratory burst function (8). The method uses a fraction of DBS corresponding to a sub-microliter volume of blood, and is applicable for population-scale performance (Figure 1).

## Methods

### Blood Samples and Preparation for Protein Profiling

Thirty-seven anonymized healthy control samples in forms of dried blood spot (DBS), serum, and whole blood (including 21 newborn samples) were provided by the Karolinska University Hospital Huddinge (Sweden) for normal range identification of the targeted proteins (see Antibodies and Immunoassay Procedure sections). Moreover, 41 DBS and matched serum samples (including five original Guthrie cards from the Newborn Screening Laboratory, Karolinska University Hospital, Sweden) with defects in one of the proteins C1, C2, C3, C4, C5, C6, C7, C8, C9, FB, FD, FH, FI, properdin, or HAX1 were obtained and prepared for retrospective screening (Table 1). All human samples used in this study were collected after informed consent and handled under the approval of the Regional Ethical Review Board (EPN) in Stockholm, Sweden.

**Table 1:** Information on the sample cohort used in retrospective screening.

**Diagnosed PID/disorder**	**Number of patient samples**	**Previous citation (if available)**	**Provided by**
C1 deficiency	1^#^		Sigma-Aldrich, product# 234401
C2 deficiency	8 (five newborns)^*^	(10, 11)	Vall d'Hebron University Hospital, Spain Son Espases University Hospital, Spain 12 Octubre Hospital, Spain Karolinska University Hospital, Sweden
C3 deficiency	2^#^	(10)	Sigma-Aldrich, product# C8788 Karolinska University Hospital, Sweden
C4 deficiency	1^#^	(10)	Karolinska University Hospital, Sweden
C5 deficiency	1	(12)	Vall d'Hebron University Hospital, Spain
C6 deficiency	2		Sigma-Aldrich, product# C1288 La Paz University Hospital, Spain
C7 deficiency	3	(13)	Son Espases University Hospital, Spain Sahlgrenska University Hospital, Sweden National Defense Medical College, Japan
C8 deficiency	1^#^		Sigma-Aldrich, product#C1538
C9 deficiency	4	(14)	Fukuoka Children's Hospital, Japan National Defense Medical College, Japan
FB deficiency	1	(15)	Royal Melbourne Hospital, Australia
FD deficiency	3	(16)	Sint Antonius Hospital, the Netherlands
FH deficiency	2^#^	(10)	Karolinska University Hospital, Sweden
FI deficiency	8	(17, 18)	Vall d'Hebron University Hospital, Spain Royal Melbourne Hospital, Australia San Pedro de Alcántara Hospital, Spain 12 Octubre Hospital, Spain
Properdin deficiency	2		Royal Melbourne Hospital, Australia Central Hospital, Kristianstad, Sweden
SCN (HAX1 deficiency)	2	(19)	Tehran University of Medical Sciences, Iran
**Total number of patients**	41		See above
**Healthy Controls**	37 (21 newborns)		Karolinska University Hospital, Sweden

* *Original Guthrie cards from newborn screening program in Sweden*.

# *Available sample type is serum*.

Filter papers (PerkinElmer) for DBS sampling were provided by the Newborn Screening Laboratory at the Karolinska University Hospital Solna (Sweden), and were prepared by applying 70 μL (corresponding to one drop) fresh and intact whole blood onto the filter paper. Papers were dried at ambient temperature (25°C) for 4 h, before desiccated storage at 4°C until use. DBS samples were punched in 3.2 mm in diameter discs (corresponding to 4.5% fraction of one blood drop), and proteins were eluted by submerging the disc in 30 μL sterile phosphate buffered saline supplemented with Tween 20 (PBS-T, pH 7.4, 0.1% Tween 20, Medicago-AB) for 3 days, 1 day, or 2 h at 4°C. Subsequently, proteins from the DBS eluate or 3 μL matched whole blood were biotinylated (referred as protein labeling) by addition of ten times molar excess of Sulfo-N-Hydroxysuccinimide-polyethyleneglycol biotin (NHS-PEG4-Biotin, ThermoFisher Scientific, USA) in 30 μL PBS-T (referred as labeling solution), with incubation for 2 h at 4°C. Alternatively, a rapid and simultaneous protein elution and labeling was performed by submerging the DBS disc in 30 μL of labeling solution for 2 h, 30 min, or 10 min at ambient temperature. Labeled samples were stored at −20°C until use. Immediately before incubation with the suspension bead array, 1 μL of labeled samples were further diluted 50 times in PBS buffer supplemented with polyvinylpyrrolidone, polyvinyl alcohol, and casein (Sigma-Aldrich). The diluted samples were heat-treated at 56°C for 30 min for signal enhancement (10) and were cooled down to ambient temperature for 10 min prior use. The effect of heat-treatment on heat-labile complement proteins with no effect on protein profiling were previously described (10). The final 1:500 dilution of eluted samples from each disc provides enough volume for 30 tests, which corresponds to a 0.1 μL volume of crude whole blood applied to each multiplexed measurement.

### Antibodies and Immunoassay Procedure

Antibodies targeting the proteins and protein fragments C1qA, C1qB, C1s, C2b, C3, C3a, C4A, C4B, C5, C6, C7, C8A, C8B, C9, FB, FD, FH, FI, Properdin, CSF3R, p22-phox, gp91-phox, p47-phox, p67-phox, p40-phox, ELANE, HAX1, human albumin and human IgG were covalently coupled to carboxylated fluorescent-color-coded Luminex MagPlex microspheres (Luminex Corporation) according to a previously established protocol (20). In brief, 500,000 microspheres (referred to as beads) were functionalized using N-Hydroxysuccinimide-polyethyleneglycol (NHS) and 1-Ethyl-3-(3-dimethylaminopropyl) carbodiimide (EDC) chemical linkers (ThermoFisher-Scientific) according to the manufacturer's protocol, and incubated with 1.65 μg of the antibody or recombinant protein in 100 μL 2-(N-Morpholino) ethanesulfonic acid (MES) buffer at pH 5.0 (Sigma-Aldrich) for 2 h at ambient temperature on a microplate shaker (650 rpm). Remaining active groups on the beads were quenched by an overnight incubation in 50 μL blocking reagent for ELISA (Roche). Antibody-coupled beads were pooled in suspension to be used as the suspension bead array in subsequent experiments. An array of 42 antibodies targeting the 22 proteins and their corresponding protein fragments was used for protein profiling (Table S1).

Fifty microliter of labeled, diluted, and heat-treated samples were applied to the prepared suspension bead array with bead counts of minimum 30 beads per target per measurement. After overnight incubation in a sealed plate at ambient temperature on a microplate shaker (650 rpm), the beads were washed three times in 100 μL PBS-T. The sandwich immunoassay was performed as described above, with the exception of using unlabeled DBS eluate, and addition of an incubation step with biotinylated secondary antibodies for 1 h at ambient temperature, followed by three times washes in 100 μL PBS-T (21). Subsequently, the captured biotin-labeled proteins on beads (or detection antibodies in sandwich format) were incubated with 50 μL of Streptavidin R-phycoerythrin conjugate (SAPE, ThermoFisher-Scientific) at the concentration of 5 μg/mL. After three times washes in 100 μL PBS-T and resuspension in 100 μL PBS-T, fluorescent signal intensities were measured in parallel for all targets using Luminex LX200 and FLEXMAP 3D systems (Luminex Corporation) according to the manufacturer's instructions.

### Data Analysis

R software version 3.2.3 and R Studio environment version 0.99.902 were applied for statistical analyses and data visualization (22). The median fluorescence intensities (MFI) from the beads regarding each protein target per measurement were used. The signal-to-background ratio was determined according to the internal negative control (data from beads with no capture antibody). Data were normalized and fluorescent signals were adjusted using the “batch effect removal” function from the R Limma package (23). The statistical cut-off for the definition of protein defects (PID level) was considered as an outlier fluorescent intensity under minimum values that deviates from the 1.5 times interquartile range (IQR) under the first quartile of corresponding signals for each protein measurement in all healthy control samples. Values from PID patients associated with non-targeted proteins were not considered as healthy values, as no previous clinical data is available for non-targeted proteins and deficiency in one complement protein can cause perturbation in the levels of other proteins due to regulatory, compensation and feedback mechanisms. The coefficient of variation (CV) was calculated for the assay as the ratio of the standard deviation to the mean for triplicate measurements from three different samples, two different sample material and 43 antibodies.

## Results

Initially, we confirmed protein elution from filter paper of three healthy controls, evaluated the protein measurements, as well as testing automation and reduction in turnaround time (Figure S1A–C). The procedure was set to 30 min protein elution and labeling at ambient temperature, followed by an overnight protein capture on an antibody-coupled bead array, and parallel fluorescent-based readout for 30 s per sample on the following day. The median coefficient of variation (CV) of the method was calculated to be 15. An optimal limit for blood dilution of above 1:800 was preferred to allow the detection of as many proteins as possible.

Subsequently, screening was performed on a sample cohort, including 37 healthy controls and 41 cases diagnosed with PID (Table 1). The cohort included 26 newborn samples, including five PID cases of original Guthrie cards from the Newborn Screening Laboratory at Karolinska Hospital (Sweden) and 21 healthy DBS samples provided by the National Cord Blood Biobank at Karolinska University Hospital Huddinge (Sweden). Additionally, these five original Guthrie card samples from newborns with PID were compared with healthy DBS samples for confirmation of the newborn screening concept.

**Figure 2:**
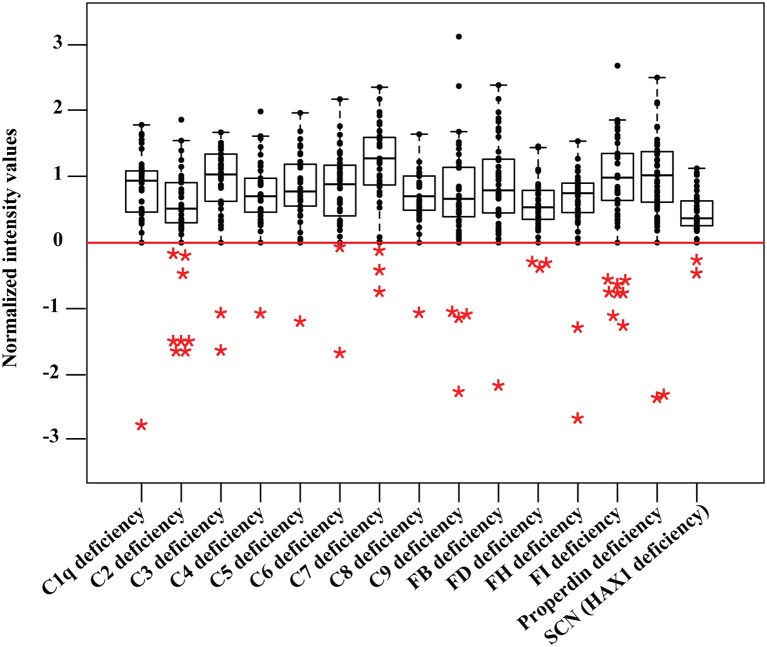
Data from retrospective PID screening. Protein profiling is done in parallel in a multiplexed measurement and obtained values are shown in separate boxplots for different disorders from 37 healthy control samples including 21 newborn dried blood spots. The deficiency level (IQR ≥ 1.5) is shown as a red baseline. Deficient samples (*n* = 41) are shown in colored asterisks, including five newborn dried blood spots of original Guthrie cards from newborn screening program in Sweden. SCN stands for severe congenital neutropenia.

Samples were profiled for complement components C1-C9, complement factors FB, FD, FH, FI, and properdin, as well as the proteins related to phagocytosis and granulocyte function, including colony-stimulating factor 3 receptor (CSF3R), phagocytic oxidase subunits p22-phox, gp91-phox, p47-phox, p67-phox, and p40-phox, neutrophil elastase (ELANE) and HCLS1 associated protein X-1 (HAX1) (Table S1). The deficiency level for each protein was defined based on the population of 37 healthy samples, as those with a deviation from healthy control values within the interquartile range of 1.5 (IQR ≥ 1.5). Data obtained from the retrospective screening confirmed the documented disorders within the analyzed cohort (Figure 2, Figure S2), mainly in DBS samples collected from neonates with PID, compared to normal newborn samples or healthy adults (Table 1). Furthermore, the signals obtained from deficient samples were shown to be regained by supplementing the corresponding recombinant protein into the DBS samples collected from the respective deficient donors (Figure S1D). The second-tier screening was also shown to be accessible in the form of sandwich immunoassays, allowing for a more selective detection of the target proteins (Figure S3). Lastly, the screening data were further validated by current standard clinical tests in matched serum samples of the PID cohort (Table S2).

## Discussion

Newborn screening programs are required to meet a panel of defined criteria for the principles and practice of each diagnostic method to be considered at a public health measure. The main criteria are that the disorder needs to be a serious health issue and late-stage treatment would cause increased morbidity and mortality, while early diagnosis would lead to a significantly better outcome, a treatment or effective preventive care should be readily available for the diagnosed condition, the disorder should have a high enough incidence among the target population, the costs involved should be economically balanced to adapt for massive population screening, and the turnaround time from sampling to data should not exceed 48 h (2).

The genetic disorders of the innate immunity are very serious, particularly when considering life-threatening infections and angioedema. Late diagnosis would cause significantly higher morbidity leading to permanent damage to vital organs and even death. A strict preventive care such as prophylactic antibody therapy, preventive treatment for systemic lupus erythematosus (SLE) and HAE, as well as postponing BCG vaccination and early hematopoietic stem cell transplantation (HSCT) in CGD patients would significantly reduce morbidity and mortality. Currently, the cumulative prevalence of deficiencies of the innate immunity, which could be markedly underestimated due to lack of accurate diagnosis, is within the range for newborn screening (1 in <20,000 live births in the target population). The cost per assay for the proposed method is below 5 USD for the detection of the 22 disorders (including the costs for all reagents and laboratory consumables) and is shown to be in the range of newborn screening guidelines for massive population screening. The turnaround time from sampling to data is fewer than 10 h and fits well with the time standard.

A key benefit of the developed screening system is the notable number of PID-associated proteins that can be examined in parallel using a limited amount of sample material. Moreover, the flexible design of the bead array content enables an optimal adaptation to national programs of different countries, since the prevalence and mechanism of PIDs are markedly different among ethnic groups. Nevertheless, the presented screening might miss identifying cases of rare, newly discovered types of PIDs, or disorders caused by a dysfunctional protein expression. Updating the array to include particular antibodies that bind the mutated, truncated, or distorted protein fragments might further identify these additional PID cases. The bead array technology can increase the sample throughput up to 384 individuals per instrument run, with a readout capacity of up to over 500 proteins in parallel (10, 20). We have shown the possibility for an extended array design to reach beyond the presented 22 disorders, by profiling over 1,000 protein targets in DBS samples (Figure S4).

Taken together, combining the long-established routines for DBS neonatal sampling with the inherent potential of multiplexed technologies offers an appealing avenue that can be readily expanded to nationwide newborn screening for a broader range of PIDs. In the future perspective, a pilot program would address the aspects of population-scale screening prior to the decision of implementation in the national screening programs. In addition, the actual prevalence rates of these PIDs will be adjusted based on data from the pilot program. As an instance, the prevalence of severe combined immunodeficiency (SCID) in the US was significantly adjusted from 1 in 100,000 to 1 in 58,000 live births before and after the pilot newborn screening study (24). Although newborn screening of severe diseases like CGD is urgently required, the pilot screening will confirm the usefulness of the screening for each country and will support the governmental decisions on the set of other innate PIDs to include in the nation-wide screening program. Since our current suggested method is flexible, depending on the prevalence of PIDs in a specific country the cost-effectiveness assessments in their health-care systems can be performed to designed required custom array. It is estimated that the innate immunodeficiency screening would benefit 20 newborns per year in Sweden and 6,500 newborns worldwide.

The current study should be considered a “proof of principle” and the precursor for a pilot study with enough samples size to make a decision as to cost/benefit and feasibility. Importantly, the presented approach could pave the way for an earlier, more comprehensive and accurate diagnosis of complement and phagocytic disorders. Correct and precise diagnosis with an insight over several conditions could inform a better understanding of these diseases by finding patients with specific or combination of deficiencies, in addition to showing the differences between non-functional vs. abrogated protein expressions. With a more personalized diagnosis at hand, insights from multi-parameter assays can contribute to a more effective treatment and preventive care, which ultimately leads to a healthy and active life for PID patients.

## Data Availability Statement

The raw data supporting the conclusions of this article will be made available by the authors, without undue reservation, to any qualified researcher.

## Ethics Statement

The studies involving human participants were reviewed and approved by the Regional Ethical Review Board (EPN) in Stockholm, Sweden. Written informed consent to participate in this study was provided by the participants' legal guardian/next of kin.

## Author Contributions

MD, SB, LS, HA, MN, JS, PN, and LH conceived and designed the study. MD and SB planned and performed the laboratory experiments and acquisition of data in screening. LS designed and performed the validation. MD, SB, LS, HA, MT-R, JS, PN, and LH analyzed and interpreted the data and revised the manuscript critically. LS, HA, CF, JF, CS, AR, LF, ML-T, LG-G, LA-M, YM, YY, VF, ÅL, AA, NR, AM-N, MH-G, UD, LT, TH, SN, JS, and PN contributed with patient diagnosis and/or providing of reagents, materials, analysis tools. MD wrote the paper. All authors approved the final manuscript for submission.

### Conflict of Interest

The authors declare that the research was conducted in the absence of any commercial or financial relationships that could be construed as a potential conflict of interest.
