# Supplementary material for: Newborn Screening for Presymptomatic Diagnosis of Complement and Phagocyte Deficiencies
Source: Front Immunol. 2020 Mar 17;11:455. doi: 10.3389/fimmu.2020.00455 (PMC7090021; doi:10.3389/fimmu.2020.00455)
Supplement: Supplementary file 1 [file Data_Sheet_1.pdf]

Supplementary material

Newborn Screening for Presymptomatic Diagnosis of Complement and Phagocyte Deficiencies

Dezfouli et al.

Supplementary Results

Assessment of protein profiling in DBS samples

Protein profiling of three healthy control DBS and matched whole blood samples in three replicates showed strongly correlated signal intensities (R= 0.96) within the two sample types, confirming successful protein elution from the filter paper (**Figure S1, A)**. Equal profiles were observed for a separate labeling step (for an additional 2 hours) after 2 hours elution of proteins from the filter paper, compared to simultaneous elution and labeling (merged for 2 hours). The obtained results from four distinct antibodies confirmed the possibility of reducing the merged elution and labeling time to as little as 10 minutes, with the highest signal-to-background ratio after 30 minutes of merged elution and labeling at ambient temperature (**Figure S1, B**). A significant correlation to the sample dilution factor (R mean = 0.98) was observed in the linearity-of-dilution test and confirmed the functionality of the capture antibodies in the complex whole-blood sample context (**Figure S1, C)**. Finally, recombinant human C2 protein, spiked into eluate of a C2-deficient DBS sample, confirmed the regain of signals by the addition of the defective protein (**Figure S1, D**).

Assessment of the effect of age on protein measurements

Investigating the effect of age difference on the proteomic profiles of 6 healthy DBS samples (3 infant and 3 adult samples), no significant differences were observed between infants and adult individuals, although the measurements showed slightly lower protein amounts (on average 1:1.4) in infants versus adult individuals. This is previously described as lower

immune-related protein levels in neonates, normalized by development and activation of the innate immunity through environmental microbial encounter after birth [1].

Assessment of sandwich assays as second-tier screening

In an anticipated nation-wide screening, a more selective detection is required for a second confirmation of indistinct reports, commonly known as the second-tier assays. For this, single-target experiments in a sandwich format were designed and performed. Results showed selective signals from the corresponding capture and detection antibodies, confirming the possibility of using sandwich immunoassays as the second-tier measurement following the population-scale screening (**Figure S3**).

Assessment of expanding the bead array content

The possibility to expand the profiling in order to cover additional proteins and disorders was investigated. Suspension bead array technology was used with a collection of 2009 randomly selected antibodies to profile 1203 protein targets in three healthy control DBS samples. Measurements were done in six experiments with multiplex detections from up to 394 antibodies per assay. The results showed signal-to-noise ratio$\geq$1 for 1156 of the random targets (>96%), confirming the possibility for an extended array for analyzing DBS samples, reaching beyond the presented panel of 22 proteins (**Figure S4**).

Supplementary Tables

Table S1. Information on the protein targets, antibodies, and recombinant proteins used as capture or detection reagents in suspension bead array experiments.

**Antibody / Protein coupled to bead array**	**Provider**	**Product number/ batch**	**Related PID / Disorder ****
C1qA	R&D Systems	MAB4035/Clone394107	C1 deficiency, SLE
C1qB*	Human Protein Atlas	HPA052116	C1 deficiency, SLE
C1s	Human Protein Atlas	HPA018852	C1 deficiency, SLE
C2b*	R&D Systems	MAB1936/ Clone269716	C2 deficiency, SLE
C3	Human Protein Atlas	HPA020432	C3 deficiency LOF, GN, aHUS
C3	Biosystems International Kft	Bsi019/ 0190-2	C3 deficiency LOF, GN, aHUS
C3a*	R&D Systems	MAB3677	C3 deficiency LOF, GN, aHUS
C4A	Biosystems International Kft	Bsi0150/ 0150090310	C4 deficiency, SLE
C4A	Biosystems International Kft	Bsi0808/ 0808-2	C4 deficiency, SLE
C4B*	Biosystems International Kft	Bsi0402/ 0462100010	C4 deficiency, SLE
C4B	AVIVA Systems Biology	OALA02951	C4 deficiency, SLE
C5*	Biosystems International Kft	Bsi0765/ 0765160410	C5 deficiency
C6*	AVIVA Systems Biology	OASA01018	C6 deficiency
C7*	AVIVA Systems Biology	OASA01019	C7 deficiency
C8A	Human Protein Atlas	HPA028225	C8 deficiency
C8A*	Human Protein Atlas	HPA054317	C8 deficiency
C8B	Biorbyt	orb182656	C8 deficiency
C9*	Biosystems International Kft	Bsi0270/ 0270080320	C9 deficiency
FB*	R&D Systems	MAB2739 Clone313011	FB deficiency, aHUS
FB	AVIVA Systems Biology	OAAB05624	FB deficiency, aHUS
FD*	R&D Systems	AF1824	FD deficiency
FH	Biosystems International Kft	Bsi1328/ 1328-2	FH deficiency, aHUS
FH*	R&D Systems	MAB4779 Clone 556317	FH deficiency, aHUS
FI*	Biorbyt	orb87484	FI deficiency, aHUS
Properdin*	Abcam	ab17780	Properdin deficiency
CSF3R*	Human Protein Atlas	HPA048086	G-CSF receptor deficiency
p22-phox*	AVIVA Systems Biology	OASA02663	Autosomal recessive CGD
gp91-phox*	MBL International Corporation	D162-3 Clone7D5	X-linked CGD
ELANE*	ABNOVA	H00001991-M02	SCN1, SCN2, Cyclic Neutropenia
HAX1	R&D Systems	AF5458	SCN3
HAX1*	AVIVA Systems Biology	OAAB08569	SCN3
p47-phox *	Human Protein Atlas	HPA047836	Autosomal recessive CGD
p47-phox	Human Protein Atlas	HPA052095	Autosomal recessive CGD
p67-phox	Human Protein Atlas	HPA002327	Autosomal recessive CGD
p67-phox *	Human Protein Atlas	HPA006040	Autosomal recessive CGD
p40-phox *	Human Protein Atlas	HPA036156	Autosomal recessive CGD
Human serum albumin	Biosystems International Kft	Bsi0097/ 0097-6	positive control
Human serum albumin	Biosystems International Kft	Bsi2684/ 2684-1	positive control
human IgG	Jackson Immuno-Research Laboratories	309-005-082	positive control
beads with no antibody	-	-	negative control (bare beads BB)

** Selected antibody is shown in figure 2/ figure S2*
*** Abbreviations:*
SLE: Systemic lupus erythematosus
LOF: Loss of function
GN: Glomerulonephritis
CGD: Chronic granulomatous disease
SCN: Severe congenital neutropenia
G-CSF: Granulocyte colony-stimulating factor
aHUS: Atypical hemolytic uremic syndrome

Table S2. Data from standard clinical tests, validating the results from retrospective screening assay.

**Defective protein**	**Number of samples** ##	**Value**	**Normal range**	**Method**
C2	3	<6 %	77-159	Rocket immunoelectrophoresis
		<6 %		
		<6 %		
C3	1	<0.06 g/L	0,77-1,38	Nephelometry
C5	1	<3 %	72-171	Rocket immunoelectrophoresis
C6	2	<6 %	63-154	Rocket immunoelectrophoresis
		<6 %		
C7	3	<6 %	64-154	Rocket immunoelectrophoresis
		<6 %		
		<6 %		
C8	1	<6 %	45-203	Rocket immunoelectrophoresis
C9	4	not detectable	Qualitative test	Gel diffusion
		not detectable		
		not detectable		
		not detectable		
FB	1	<3 %	59-154	Rocket immunoelectrophoresis
FD	3	<12.5 %	75-141	Hemolytic gel assay
		<12.5 %		
		<12.5 %		
FI	8	<5 %	60-152	Rocket immunoelectrophoresis
		<5 %		
		<5 %		
		<5 %		
		<5 %		
		<5 %		
		<5 %		
		<5 %		
Properdin	2	<6 %	54-157	Rocket immunoelectrophoresis
		<6 %		

## Only samples with available matched serum at sufficient volumes were included for validation with standard tests.

Supplementary Figures

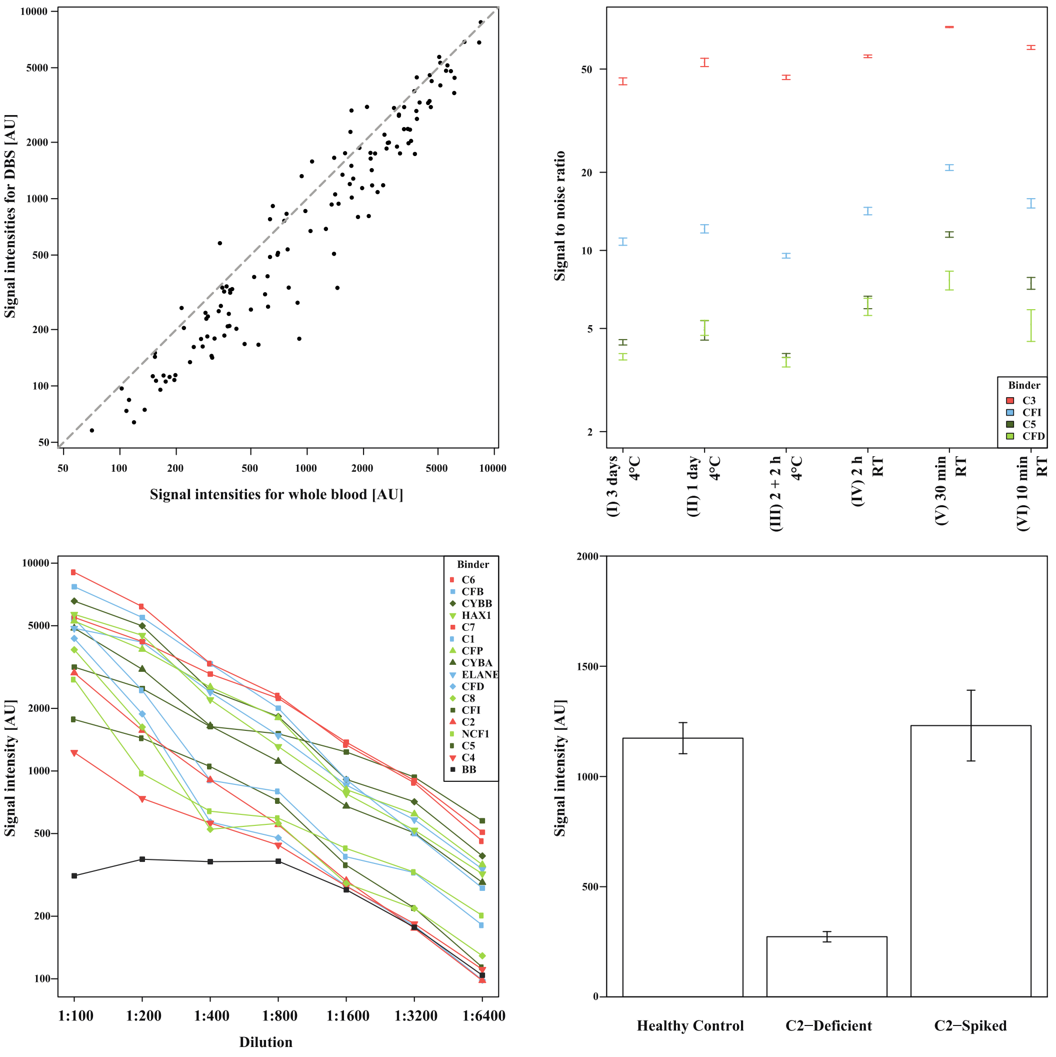

Figure S1. Data from DBS protein elution and profiling on suspension bead arrays.

A) Protein profiling of blood eluted from filter papers shows a significant correlation to the values from matched whole blood ($r^2 = 0.96$). The median coefficient of variation (CV) of the method was calculated to be 15. **B)** Simultaneous elution and labeling for 30 min at ambient temperature (RT) shows the highest signal-to-background ratio (V). No significant difference is observed for simultaneous elution and labeling (IV) versus separate labeling and elution steps (III). **C)** Linearity-of-dilution test shows significant correlation of signal intensities to the dilution factor ($r^2 = 0.98$) indicating that sample analysis above 1:800 are preferred to allow the detection of as many proteins as possible. **D)** Spiked human C2 recombinant protein into C2-deficient sample confirms the regain of the signal by the addition of the defective protein.

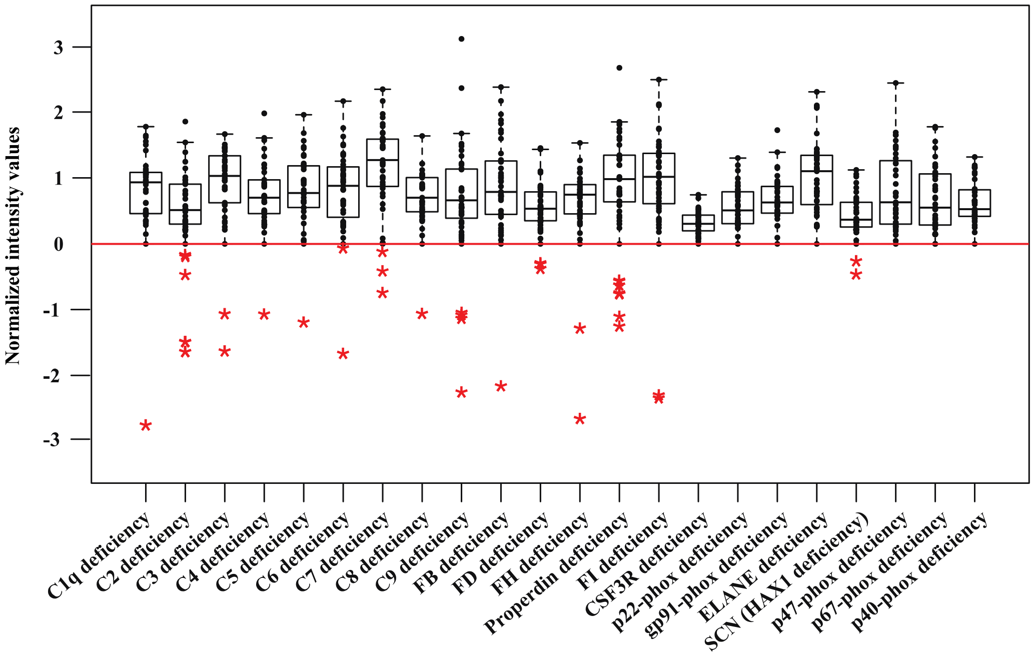

Figure S2. Data from retrospective PID screening. Protein profiling is done in multiplexed and obtained signals for each protein are shown in separate boxplots. Deficiency level (IQR≥1.5) is shown as a red baseline. Deficient samples (n=41) are shown in colored asterisks. All samples within the analysed cohort were healthy for the expression of proteins CSF3R, ELANE, p22-phox, gp91-phox, p47-phox, p67phox, and p40-phox.

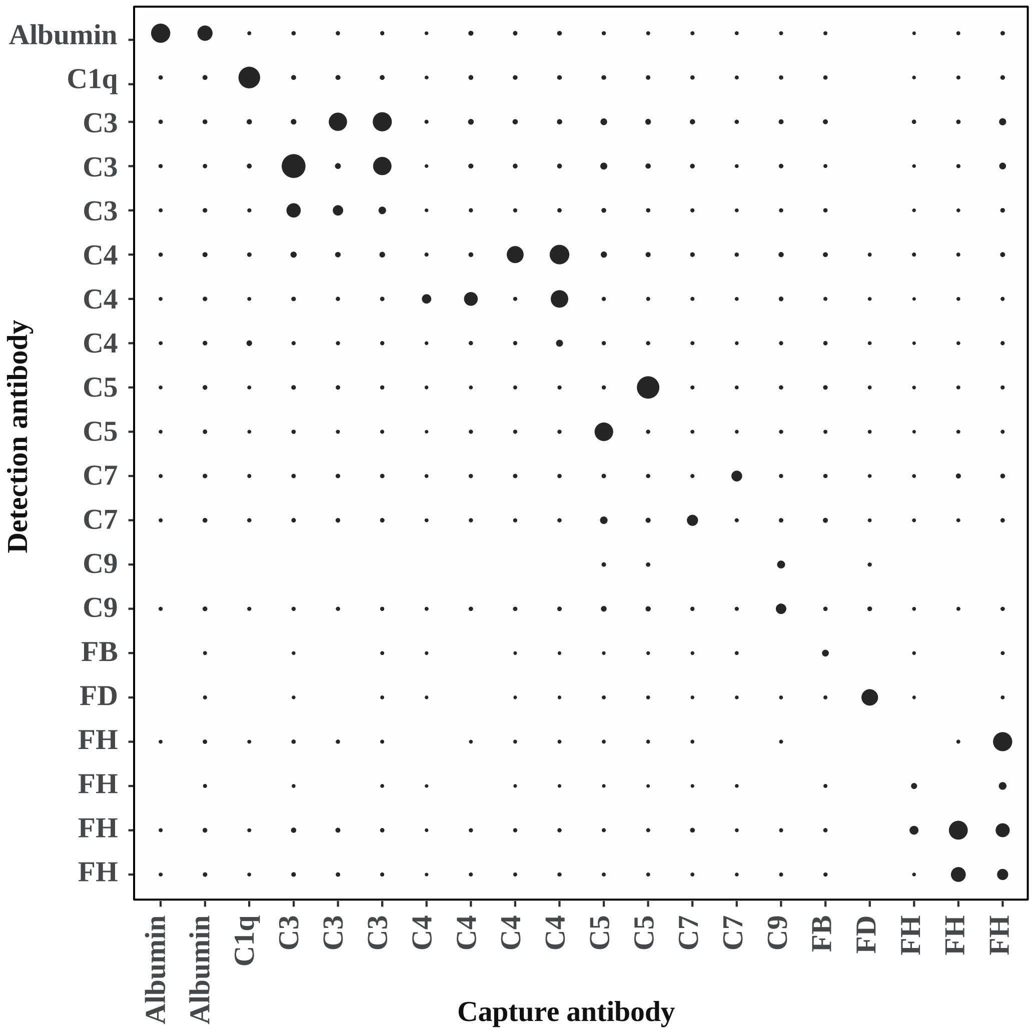

Figure S3. Data from second-tier sandwich immunoassays. Signal intensities (shown by the diameter of the circles) illustrate selective detections from the correct combination of capture and detection antibodies.

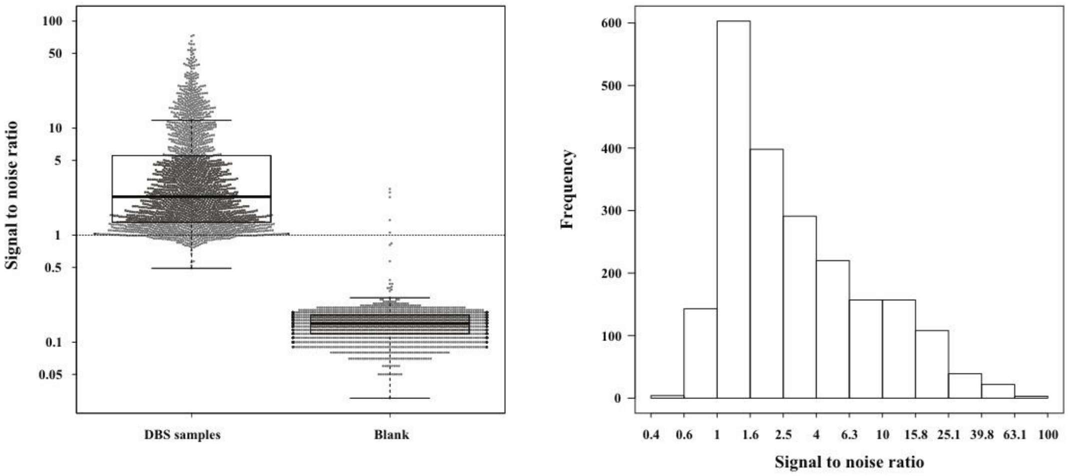

Figure S4. Data from profiling of 1203 proteins in DBS eluate. The results confirm the signal-to-noise ratio over the background level (shown in dashed line) for 1156 proteins. This confirms the possibility for an extended array design, when additional protein targets might be required for an updated screening panel.

Supplementary references

1 Grumach AS, Ceccon ME, Rutz R, Fertig A, Kirschfink M. Complement profile in neonates of different gestational ages. Scandinavian journal of immunology. 2014 Apr;79(4):276-81.
